# pH-responsive theranostic nanocomposites as synergistically enhancing positive and negative magnetic resonance imaging contrast agents

**DOI:** 10.1186/s12951-018-0350-5

**Published:** 2018-03-27

**Authors:** Xi Huang, Yaping Yuan, Weiwei Ruan, Lianhua Liu, Maili Liu, Shizhen Chen, Xin Zhou

**Affiliations:** State Key Laboratory of Magnetic Resonance and Atomic and Molecular Physics, Wuhan Institute of Physics and Mathematics, Chinese Academy of Sciences; Wuhan National Laboratory for Optoelectronics, Wuhan, 430071 China

**Keywords:** Theranostic nanoprobe, T_1_/T_2_ dual-mode, Magnetic resonance imaging, Contrast agents, Lung cancer

## Abstract

**Background:**

The rational design of theranostic nanoprobe to present responsive effect of therapeutic potency and enhanced diagnostic imaging in tumor milieu plays a vital role for efficient personalized cancer therapy and other biomedical applications. We aimed to afford a potential strategy to pose both T_1_- and T_2_-weighted MRI functions, and thereby realizing imaging guided drug delivery and targeted therapy.

**Results:**

Theranostic nanocomposites Mn-porphyrin&Fe_3_O_4_@SiO_2_@PAA-cRGD were fabricated and characterized, and the nanocomposites were effectively used in T_1_- and T_2_-weighted MRI and pH-responsive drug release. Fluorescent imaging also showed that the nanocomposites specifically accumulated in lung cancer cells by a receptor-mediated process, and were nontoxic to normal cells. The r_2_/r_1_ ratio was 20.6 in neutral pH 7.4, which decreased to 7.7 in acidic pH 5.0, suggesting the NCs could act as an ideal T_1_/T_2_ dual-mode contrast agent at acidic environments of tumor. For in vivo MRI, T_1_ and T_2_ relaxation was significantly accelerated to 55 and 37%, respectively, in the tumor after i.v. injection of nanocomposites.

**Conclusion:**

The synthesized nanocomposites exhibited highly sensitive MRI contrast function no matter in solution, cells or in vivo by synergistically enhancing positive and negative magnetic resonance imaging signals. The nanocomposites showed great potential for integrating imaging diagnosis and drug controlled release into one composition and providing real-time imaging with greatly enhanced diagnostic accuracy during targeted therapy.
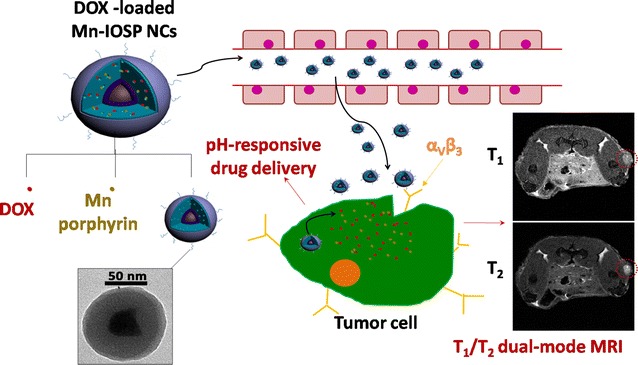

**Electronic supplementary material:**

The online version of this article (10.1186/s12951-018-0350-5) contains supplementary material, which is available to authorized users.

## Background

The magnetic resonance imaging (MRI) technique has been introduced to clinic to provide multiplanar imaging for the soft tissues in the body without invasion. Because of its superb soft tissues imaging contrast, multidimensional imaging function, and absent of ionizing radiation, MRI is becoming increasingly available for clinical imaging [1]. In 2016, there were about 39 million diagnostic MRI procedures carried out in the United States, which presents an average annual growth rate of around 4% during the past 5 years [2].

Globally, contrast agents (CAs) are widely employed in the MRI, for which over 200 million doses had been administered [3]. The most frequently used reagents for contrast enhancement are gadolinium-based, such MRI contrast agents are representative T_1_ contrast agents that can effectively curtailing the T_1_ relaxation time of protons inside tissues by interactions with the neighboring contrast agent [4]. All clinically approved Gd-CAs are small molecules. For the reduction of toxicity, Gd^3+^ ions are usually chelated using DOTA (1,4,7,10-tetraazacyclododecane-1,4,7,10-tetraacetic acid) or DTPA (diethylene triamine pentaacetic acid) molecules [5]. Large amounts of Gd^3+^ ion chelates are injected during the clinical MRI procedure to yield millimolar concentrations required for the detection. On the other hand, nano-based CAs can be manufactured in small enough sizes to reach remote regions while simultaneously enabling surface functionalization that yields good biocompatibility, specific targeting, prolonged blood circulation time, and improved imaging effect as well as therapeutic efficacy [6–10].

Compared to traditional contrast agent, dual-mode T_1_/T_2_ MRI contrast agent can provide more accurate and detailed information associated with disease than single mode MRI contrast agent [11–20]. The dual-mode T_1_/T_2_ MRI contrast agent has gained much attention, since it can give more precise and reliable diagnostic information by the enhanced contrast effects in both T_1_ imaging with high tissue resolution and T_2_ imaging with high feasibility on detection of a lesion [15]. Further, different from other multimodal imaging technologies (e.g., MR/optical, MR/PET) [21–23], dual-mode T_1_/T_2_ MRI can provide simultaneously imaging by adopting a single instrumental system, which could avoid the differences in penetration depths and spatial/time resolutions from multiple imaging devices [24].

However, the realization of dual-mode T_1_/T_2_ contrast agents has been challenging [12]. When combining the T_1_ and T_2_ CAs together, the strong magnetic coupling between them could perturb the relaxation effect of the paramagnetic T_1_ contrast agent, resulting in undesirable quenching of magnetic resonance signal [10]. To circumvent this problem, we have rationally constructed dual-mode T_1_/T_2_ CAs with releasable T_1_ contrast materials in the weak acidic tumor microenvironments. Therefore, the distance between T_1_ and T_2_ contrast materials could be increased after responsive releasing of T_1_ contrast materials to avoid the disturbance between them. As for the microenvironment in malignance, it is distinctly different from the normal tissues for several dimensions, which owes to the alterations in the metabolism of therioma [25]. The accumulation of lactic acid and H^+^, which is related to the upregulated anaerobic glycolysis in tumorigenesis and the excessive transport of hydrogen ions from tumor cells to the outside, leads to the declined extracellular pH values and the acidic microenvironment in tumor tissue. The variation in the pH has been proved to be able to serve as an effective design for cancer diagnosis and treatment by many previous researches [26–29].

**Scheme 1:**
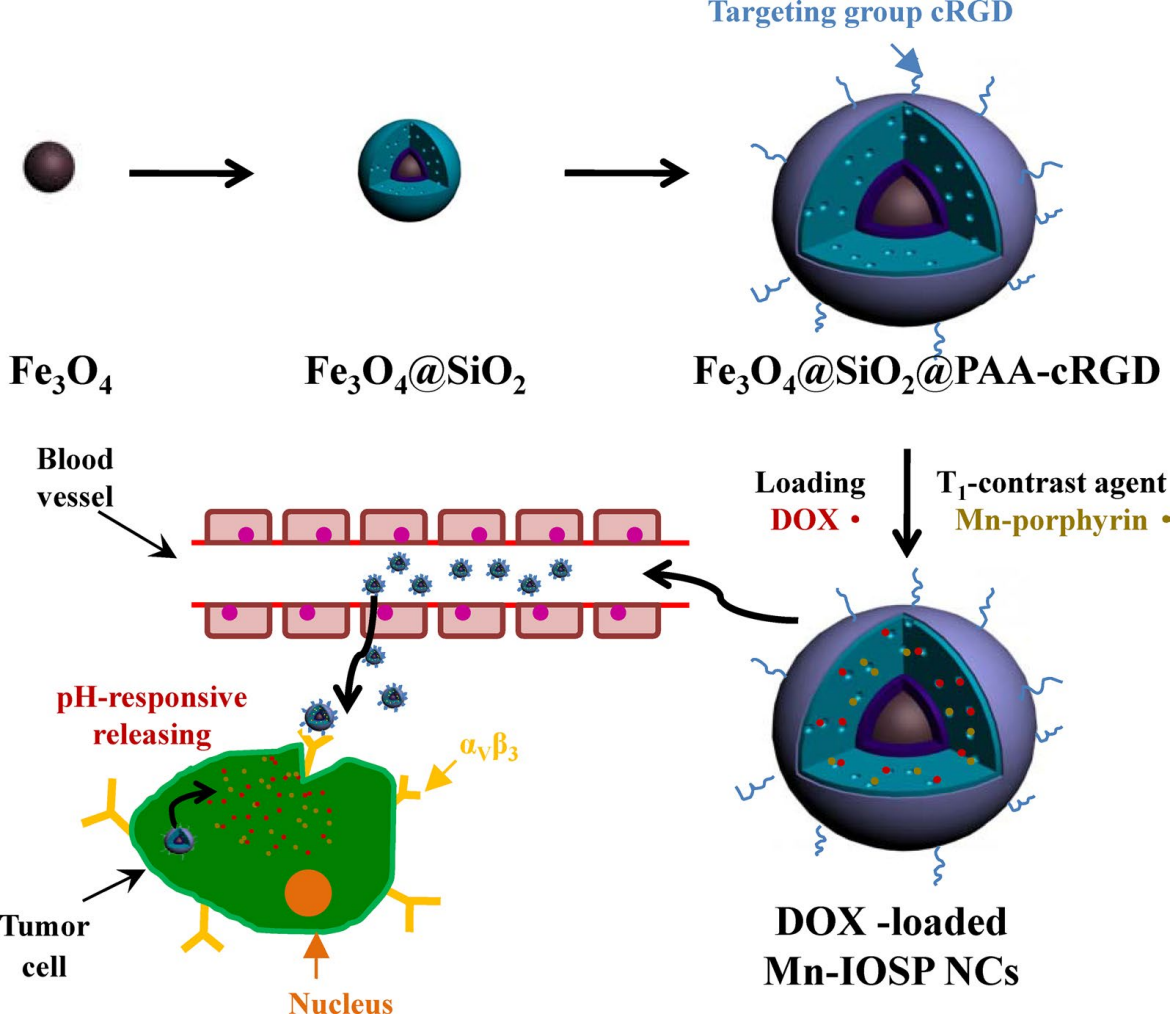
Schematic illustration for the formation, release and imaging process of DOX-loaded Mn-IOSP NCs

Therefore, we have designed a novel pH-responsive theranostic nanoprobe with Fe_3_O_4_ nanoparticles in core (T_2_ contrast agent) and water soluble Mn-porphyrin (T_1_ contrast agent) in shell, which present MRI signal “OFF” in the normal tissue and turn both T_1_ and T_2_ signal “ON” in the acidic tumor tissue (Scheme 1). The PAA coated SiO_2_ layer can be used as a pH-responsive vehicle for loading T_1_-contrast agent and anticancer drug DOX, and c(RGDyK) was chosen as the targeting group. The distance between Mn-porphyrin and Fe_3_O_4_ nanoparticles could be increased after responsive-releasing of Mn-porphyrin to avoid the disturbance between T_1_ and T_2_ contrast agents. Such design of Mn-porphyrin&Fe_3_O_4_@SiO_2_@PAA-cRGD nanocomposites (designated as Mn-IOSP NCs) affords a potential strategy to pose T_1_- and T_2_-weighted MRI functions, and thereby realizing imaging guided drug delivery and targeted therapy.

## Methods

### Reagents

Poly(acrylic acid) (PAA, Mw = 2000), *N*-hydroxysuccinimide (NHS), and 1-(3-dimethylaminopropyl)-3-ethyl carbodiimide hydrochloride (EDCI) were obtained from Aladdin. Tetraethyl orthosilicate (TEOS) and Igepal CO-520 were purchased from Sigma-Aldrich. 4-dimethylaminopyridine (DMAP), manganese (II) acetate, *N*-cetyltrimethylammonium chloride (CTAC) and triethanolamine (TEA) were purchased from Sinopharm Chemical Reagent Co., Ltd. c(RGDyK) peptides (cRGD) was purchased from GL Bioche. All other reagents were analytical grade and used without further purification.

### Characterization

^1^H and ^13^C NMR spectra for compounds were obtained in DMSO-*d*_*6*_, using a Bruker AMX-500 NMR spectrometer. Transmission electron microscopy (TEM) images were obtained on a HITACHI H-7000 FA transmission electron microscope. High-resolution mass spectrometry (HR MS–ESI) spectra were recorded on a Bruker micro TOF-Q instrument. The magnetic properties were measured at 300 K with a vibrating sample magnetometer (SQUID-VSM, Quantum Design, American). In vitro fluorescence images of cells were recorded on a confocal laser scanning microscope (CLSM, Nikon, Japan). The surface areas were measured by an ASAP-2020 physisorption apparatus (Micromeritics, American). The UV–Vis absorption spectra were determined by an Evolution 220 spectrophotometer (Thermofisher Scientific). The size distributions and zeta potentials were measured by a Malvern Zetasizer 90. The metal contents in cells and tissues were tested by ICP-MS (FLEXAR NEXLON300X).

### Preparation of Mn-porphyrin&Fe_3_O_4_@SiO_2_@PAA-cRGD (Mn-IOSP)

Fe_3_O_4_ NPs were prepared by a thermal decomposition reaction with ferric oleate complex as precursor, oleic acid as reductant, and trioctylamine as solvent at a reaction temperature of 340 °C [30]. 1.8 g of ferric oleate and 0.624 g of oleic acid were dissolved in the trioctylamine (10 mL). The mixture was heated gradually from room temperature to 340 °C and kept in this temperature for 1 h in N_2_ atmosphere. Fe_3_O_4_@nSiO_2_ was synthesized through a typical water-in-oil microemulsion method by using nonionic surfactant Igepal CO-520 [31]. For the synthesis of Fe_3_O_4_@nSiO_2_@mSiO_2_ [32], Fe_3_O_4_@nSiO_2_ was added into aqueous solution containing CTAC and TEA and stirred to be well-mixed, then TEOS was added into the solution and the hydrolysis reaction was carried out. After the reaction, the nanoparticles were purified by repeated washing and reprecipitation. For preparation of Fe_3_O_4_@SiO_2_@PAA-cRGD, we first synthesized PAA-cRGD. Then the Fe_3_O_4_@SiO_2_@PAA-cRGD NPs were synthesized from the change of the interfacial energy between PAA, Fe_3_O_4_@SiO_2_ NPs and the solvent [33]. Detailed experimental steps were presented in Additional file 1: S1.

The manganese porphyrin compounds 1 (Mn-porphyrin) was synthesized following the reported literature with some modifications [34]. Tetrakis (4-carboxyphenyl) porphyrin (TCPP, 94 mg) was mixed with EDCI (143 mg) and NHS (90 mg) in DMF and then stirred under nitrogen. After 1 h, a DMF solution containing N-boc ethylenediamine (151 mg) and DMAP (118 mg) was added into the mixture above-mentioned and continued for 1-day stir. The mixture was dealt with 100 mL brine before filtering. The N-Boc protected porphyrin (5, 10, 15, 20-tetrakis 2-[(4-tert-butyl benzamido)] ethyl carbamate] porphyrin) was separated with impurities through column chromatography using a mobile phase of DCM: methanol 90:10. The product was mixed with excess manganese acetate in methanol and reacted at 70 °C for 12 h with stirring. The N-Boc protected Mn-BOC-porphyrin (manganese 5, 10, 15, 20-tetrakis 2-[(4-tert-butyl benzamido) ethyl carbamate] porphyrin) was also purified through column chromatography using a mobile phase of DCM:methanol 90:10. Then the N-Boc protected Mn-BOC-porphyrin (30 mg) was added into CH_2_Cl_2_ with stirring for 30 min under 0 °C. HCl (4 M, 0.5 mL in dioxin) was dripped into the mixture before a 12-h stir, and the solution kept 0 °C for the duration of dripping. Then the reaction product was poured into diethyl ether and filtered. The precipitate was washed by some diethyl ether before collecting. The ^1^H NMR, ^13^C NMR, MS, UV spectra and structures for porphyrin-compounds were showed in Additional file 1: S2, including the Figs. S1–S11.

For the preparation of Mn-IOSP, 5 mg of Fe_3_O_4_@SiO_2_@PAA NPs were dissolved in a mixed solution containing 5 mL water and 45 mL isopropanol. Mn-porphyrin aqueous solution (1 mL, 10 mg/mL) was mixed into the solution before being shaken for 24 h. Finally, the desired Mn-IOSPs were gained through centrifugation separation and purification with PBS buffer.

### In vitro cytotoxicity test

The A549 non-small-cell lung cancer cells were seeded into 96-well plates for 5000 cells/well before 1-day culture. For the following 4-h incubation, the medium was changed to RPMI-1640 mediums dispersing Fe_3_O_4_@SiO_2_@PAA NCs, Mn-IOSP NCs or DOX loaded NCs with pre-established concentrations, respectively. The NCs solution was then sucked out and unabsorbed NCs were removed by rinsing with fresh PBS twice. For an additional 44-h culture, 100 μL of medium without NCs was added into every plate. The cell viabilities were tested by MTT assays with a plate reader (Molecular Devices, USA). The cell viabilities were calculated basing on the ratio of absorbance value of treatment group to that of control group. Detailed cell culture process was showed in Additional file 1: S3.

### In vivo MRI

Transplantation tumor models of non-small-cell lung cancer were established on the 4- to 5-week-old BALB/c male nude mice through subcutaneous injection of A549 cells. The mice were reared for another 2 weeks after subcutaneous injection of 1 × 10^7^ A549 cells per mouse in hind leg. T_1_- and T_2_-weighted MR images were performed on a 7.0 T animal MRI scanner (Bruker BioSpec 70/20 USR). Mn-IOSP NCs were intravenous injected into mice, and images were acquired both before and after injection. Specific parameters for images were showed in Additional file 1: S4.

### In vitro MR contrast properties

A549 cells were used for validating the in vitro MRI capacity of the Mn-IOSP NCs. After 1-h incubation with the indicated concentrations of Mn-IOSP NCs, the NCs solution was then sucked out and unabsorbed NCs were removed by rinsing with fresh PBS. Then the cells were digested and then collected with 500 μL PBS. T_1_- and T_2_-weighted MR images were performed on a 9.4 T NMR spectrometer (Bruker Avance 400, Ettlingen, Germany) equipped with microimaging gradient coils. Specific parameters were showed in Additional file 1: S4.

To measure the longitudinal (r_1_) and transverse (r_2_) relaxation parameters of Mn-IOSP NCs, different concentration points of Mn-IOSP NCs were dispersed in different buffer solutions (pH 7.4, 6.5, 5.0). T_1_- and T_2_- relaxation times of the solutions were tested by the 7.0 T animal MRI scanner using the same sequences as in vivo MRI after 1-day incubation at 37 °C.

## Results and discussion

### Fabrication and characterization

**Fig. 1:**
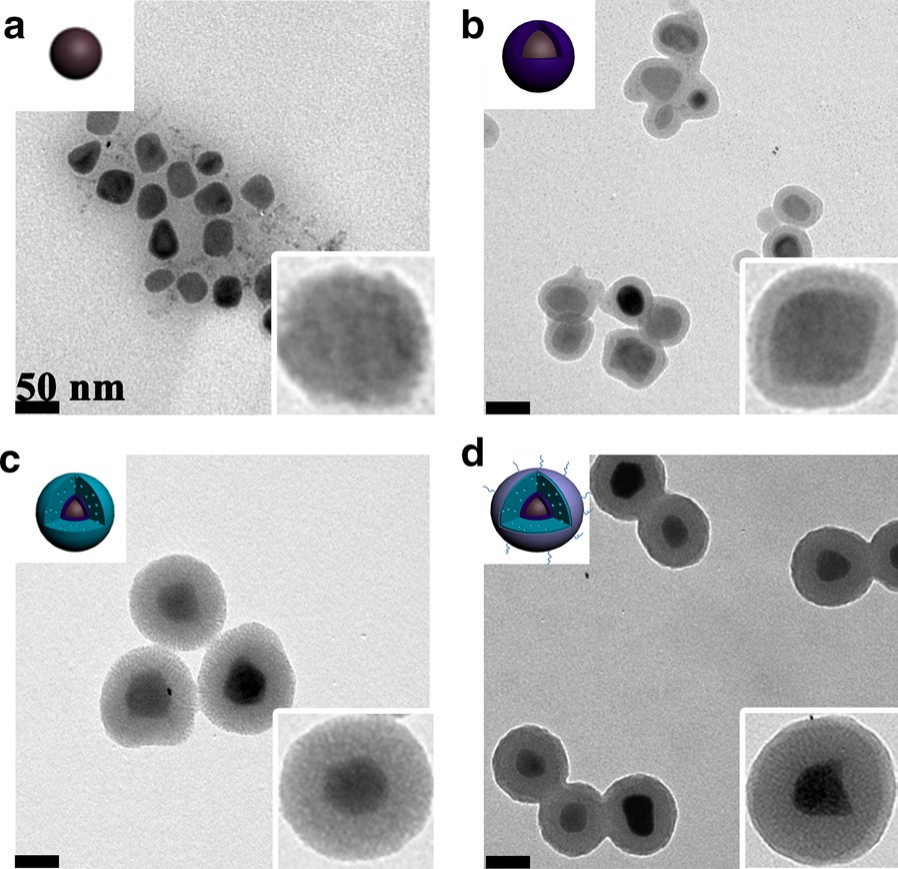
TEM images for nanoparticles: **a** OA-Fe_3_O_4_ NPs, **b** Fe_3_O_4_@nSiO_2_ NPs, **c** Fe_3_O_4_@nSiO_2_@mSiO_2_ NPs, **d** Fe_3_O_4_@SiO_2_@PAA-cRGD NPs. The scale bars are 50 nm

**Fig. 2:**
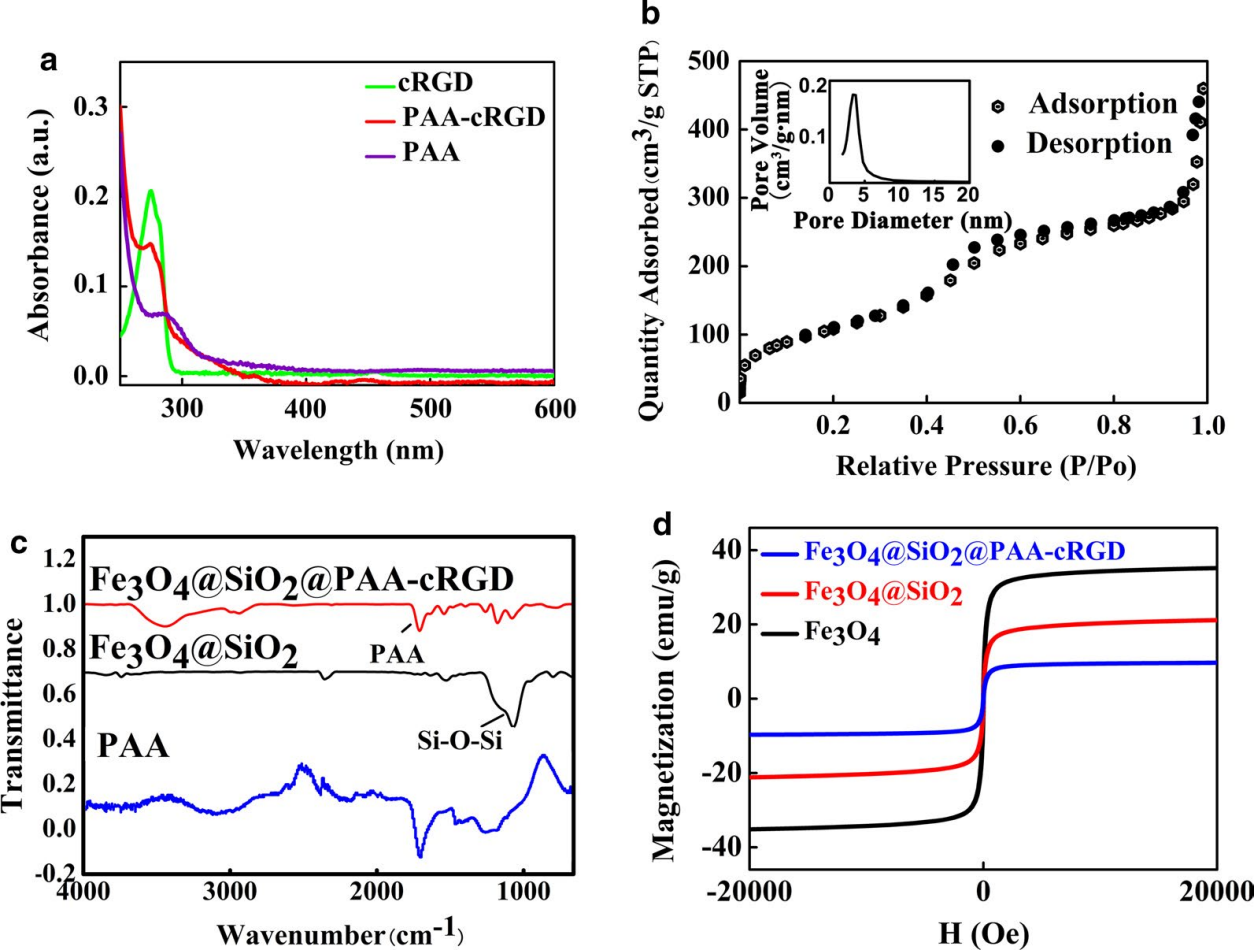
Characterization of nanoparticles: **a** UV–Vis absorption spectra of cRGD, PAA-cRGD and PAA in water, **b** N_2_ adsorption (hollow)–desorption (solid) isotherms (inset: pore size distribution from adsorption branch) of Fe_3_O_4_@SiO_2_@PAA-cRGD NPs, **c** FT-IR spectra of Fe_3_O_4_@SiO_2_ NPs, Fe_3_O_4_@SiO_2_@PAA-cRGD NPs and PAA, **d** Magnetization vs. applied magnetic field for Fe_3_O_4_, Fe_3_O_4_@SiO_2_, and Fe_3_O_4_@SiO_2_@PAA-cRGD NPs at 300 K

The formation of the multifunctional magnetic NCs could be divided into 4 steps: (1) the synthesis of the OA-coated Fe_3_O_4_ NPs, (2) the successive formation of dense SiO_2_ and mesoporous SiO_2_ shells on OA-coated Fe_3_O_4_ NPs, (3) the coating of PAA-cRGD upon the as-synthesized Fe_3_O_4_@SiO_2_ NPs, (4) the loading of Mn-porphyrin into the NPs. Finally, the Mn-IOSPs NCs were obtained and employed as a dual modality imaging contrast agent for T_1_ and T_2_. TEM images in Fig. 1a showed the size of the initial OA-Fe_3_O_4_ NPs were around 30 nm. Then the hydrophobic NPs were coated with dense SiO_2_ to form hydrophilic Fe_3_O_4_@nSiO_2_ via a controlled sol–gel reaction (Fig. 1b). The TEOS was hydrolyzed and wrapped around the Fe_3_O_4_@nSiO_2_ to form Fe_3_O_4_@nSiO_2_@mSiO_2_ (Fig. 1c). The PAA-cRGD was synthesised successfully and validated by UV absorption spectrum (Fig. 2a). PAA-cRGD and cRGD show same typical absorption peaks at 274 nm, while PAA has no obvious absorption peaks there. The encapsulation of PAA-cRGD shells outside Fe_3_O_4_@SiO_2_ may be associated with the hydrogen bond between the carboxyl groups of PAA and the hydroxyl groups of SiO_2_, and the transformation of interfacial energy between shells, nanoparticles and solvent during the loading process, which has a tendency to cause the smallest interfacial energy [33]. We can see thin layers on the surfaces of the Fe_3_O_4_@SiO_2_@PAA-cRGD NPs (Fig. 1d), suggesting that the PAA was successfully coated on the as-synthesized NPs. The Fe_3_O_4_@SiO_2_@PAA-cRGD NPs show hydrodynamic diameters of 164.2 nm and zeta potentials of − 32.2 mV compared to the 140.0 nm and − 17 mV of Fe_3_O_4_@mSiO_2_ (Additional file 1: S5, including Table S1 and Figs. S12–S16), which also confirm the successful coating of PAA shells on silica. To verify the mesoporous properties of nanoparticles, the nitrogen adsorption/desorption isotherm of Fe_3_O_4_@SiO_2_@PAA-cRGD NPs was observed (Fig. 2b). It could be calculated that the Fe_3_O_4_@SiO_2_@PAA-cRGD NPs are with high specific surface area (SBET, 349 m^2^/g) and large cumulative pore volume (Vp, 0.712 cm^3^/g), and exhibit an intensive pore diameter peak at 3.5 nm. All these results above proved the feasibility of the Fe_3_O_4_@SiO_2_@PAA-cRGD NPs to be an appropriate carrier for T_1_-contrast agents and drugs. Figure 2c showed the FT-IR spectrum of the nanoparticles. New signals at 1708 cm^−1^ from carbonyl groups of PAA is also clearly observed in the Fe_3_O_4_@SiO_2_@PAA-cRGD NPs, which further proves the formation of PAA shell. The magnetic hysteresis curves are shown in Fig. 2d to evaluate the saturation magnetization of Fe_3_O_4_@SiO_2_@PAA-cRGD NPs. At 300 K, the neglectable remanence indicates that the NPs did not possess a net magnetic moment. The measured saturation magnetization (Ms) was ~ 9.7 emu/g, suggesting their probability for improving the effect of MRI and magnetic targeting drug delivery [35].

### In vitro fluorescence and UV–Vis analysis

The capacity of nanoparticles for loading drug and Mn-porphyrin has been evaluated. The loading efficiency and releasing rate of modal drug doxorubicin hydrochloride (DOX) for Fe_3_O_4_@SiO_2_@PAA-cRGD NPs was evaluated by fluorescence spectrophotometer at different time points emission at 590 nm and excitation at 495 nm. AAS and UV spectra were used for the quantification of Mn-porphyrin in the NCs. Detailed drug loading and release procedures were showed in Additional file 1: S6. After the loading process of the Mn-porphyrin and DOX with the nanoparticles, the desired NCs were gained through centrifugation separation and the concentrations of Mn-porphyrin and DOX in supernatant were determined. The loading efficiency of DOX could be up to 90% without adding Mn-porphyrin. The excellent efficiency was benefited from the synergistic loading function of both PAA-cRGD and mSiO_2_ shells. The loading efficiency of Mn-porphyrin in Mn-IOSP NCs calculated from UV spectra was 75%, which was approximately in accordance with the metal content ratio tested by AAS (7.4/1 for iron/manganese). The structure of Mn-porphyrin has 4 amino groups and would be easy to be positively charged in the pH from 5.0 to 7.4. The strong electrostatic attraction existed between the negatively charged PAA and silica pores and the Mn-porphyrin molecules. The possible interaction of π–π stacking between the Mn-porphyrin molecules and hydrogen bonds would make it also easy to exist in the PAA shells and silica channels. When Mn-porphyrin was added into the solution simultaneously with DOX, the loading efficiency changed to 57% for DOX and 52% for Mn-porphyrin. The loading efficiencies of both DOX and Mn-porphyrin decreased when Mn-porphyrin and DOX was added into the solution simultaneously, which indicated the competition between Mn-porphyrin and DOX in loading process for the similar mechanism in the loading process.

**Fig. 3:**
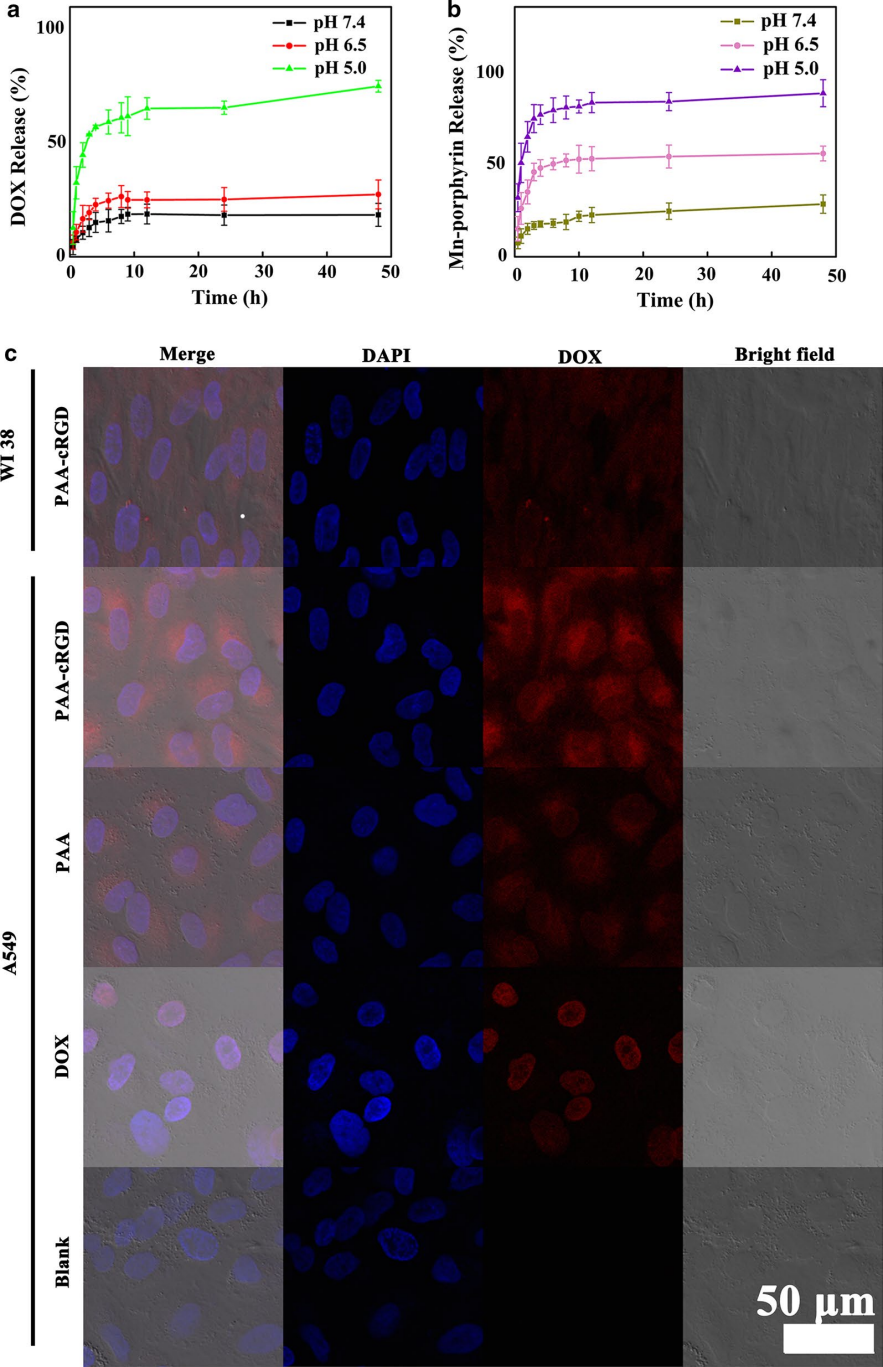
Fluorescence analysis and UV–Vis of nanoparticles: **a** pH-dependent (pH 5.0, 6.5, 7.4) drug release of DOX&Mn-IOSP NCs, **b** pH-dependent (pH 5.0, 6.5, 7.4) release of Mn-porphyrin in Mn-IOSP NCs, **c** fluorescence confocal micrographs of DOX&Mn-IOSP NCs (with or without modified with cRGD) and free DOX incubated in WI38 cells or A549 cells, the scale bars are 50 μm

For investigating the pH-triggered controlled release property of the DOX&Mn-IOSP NCs, samples were dispersed in different release media to simulate the release behavior in different physiological environments. As shown in Fig. 3a, only 18% DOX was released from DOX&Mn-IOSP NCs at pH 7.4 for a period of 48 h at 37 °C, while the release rate could reach 75% at pH 5.0, indicating the good pH sensitivity of Mn-IOSP NCs. This is because DOX is positively charged in the three pH of study, and the abundant carboxyl groups in PAA are protonated with the decreasing of pH in the medium, which weaken the electrostatic interaction with positively charged DOX [36]. For investigating the in vitro release property of the Mn-porphyrin, equal amounts of Mn-IOSP NCs were dispersed in different release media to simulate the release behavior (Fig. 3b). In the pH 5.0, the release rate could reach up to 88%, and the release has gone quickly at the first 2 h. These evidences suggest that the release rate of model molecules DOX and Mn-porphyrin from NCs might be accelerated in the mildly acidic environments of tumor areas compared to the neutral physiological environment of normal tissue. When the Mn-porphyrin released from the NCs, the Mn-porphyrin could go apart from the interference of the strong magnetic field induced by Fe_3_O_4_ core [7] and activated the T_1_ contrast ability of Mn-IOSP NCs. The ability increased with the rising release rate of Mn-porphyrin, which was along with the decrease of pH. As a result, the synthesized Fe_3_O_4_@SiO_2_@PAA-cRGD NCs and Mn-IOSP NCs can be a promising platform for pH-response MRI and drug delivery during cancer treatment.

The confocal fluorescence imaging was also employed to analyse the NCs (Fig. 3c). In the A549 cells which were incubated with DOX&Mn-IOSP NCs, strong red fluorescence was displayed in the cytoplasm and strong blue fluorescence was obtained in the nucleus. The blue fluorescence occurred by staining with DAPI and excited with a 405 nm laser, and the red fluorescence excited with a 488 nm laser from DOX indicates the successful entrance into the cell and the release of the DOX of the DOX&Mn-IOSP NCs. To validate the red fluorescence in the cell was caused from the entrance of NPs rather than only the DOX previously released, A549 cells was incubated with free DOX and both red and blue fluorescence was displayed in the nucleus, which was different from cells incubated with NPs, declaring the two different ways for the DOX coming into cells.

Tumor targeting ability of the Mn-IOSP NCs was investigated by CLSM following the incubation of A549 cells and WI38 cells with the DOX&Mn-IOSP NCs. The red fluorescence (attributed to DOX) in A549 cells incubated with DOX&Mn-IOSP NCs was distinguishably higher than that of NCs without modified with c(RGDyK), and the fluorescence intensity in A549 cells was obviously higher compared with that in non targeted WI38 cells under same incubation condition (Fig. 3c), which verified the specific targeting ability of NCs. DOX fluorescence was observed mostly in the cytoplasm instead of the cell nuclei, suggesting that DOX-loaded NCs were internalized and release DOX into the cytoplasm after uptake by A549 cells.

### Relaxivity measurements

**Fig. 4:**
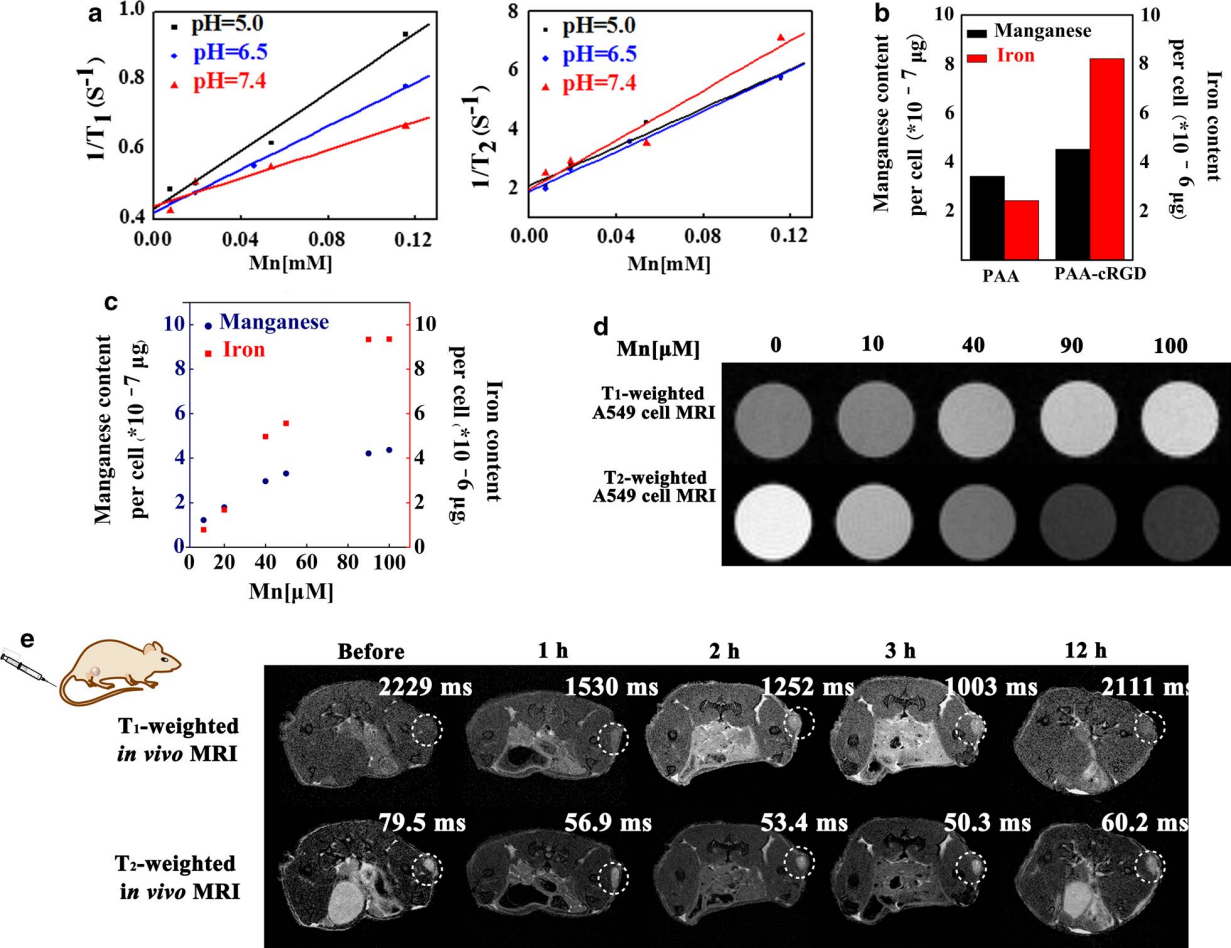
Relaxivity measurements and cell metal content determination of Mn-IOSP NCs: **a** the longitudinal relaxivity and transverse relaxivity of Mn-IOSP NCs at different pH (5.0, 6.5, 7.4), plots for 1/T_1_ and 1/T_2_ *vs.* Mn concentration, **b** iron and manganese content in A549 cells due to the uptake of NCs which modified with PAA-cRGD or PAA, **c** iron and manganese content in A549 cells due to the uptake of Mn-IOSPs in different concentrations, **d** T_1_- and T_2_-weighted MRI for A549 cells incubated with Mn-IOSP NCs at different concentrations, **e** in vivo T_1_- and T_2_-weighted MRI acquired before and after i.v. injection of Mn-IOSP NCs, the tumor sites were circled roughly with white dotted lines

To evaluate the capacity of Mn-IOSP NCs as T_1_/T_2_ dual-mode contrast agents, a 7.0 T animal MRI scanner was used and both longitudinal (r_1_) and transverse relaxivities (r_2_) were calculated. The content of iron and manganese in Mn-IOSP NCs were determined by atomic absorption spectrometry (AAS). Iron content in Mn-IOSP NCs was about 16 wt% and atomic content of iron was 7.4 times that of manganese in Mn-IOSP NCs. Samples of the Mn-IOSP NCs in different pH value of buffer containing various metal concentrations were scanned. The longitudinal relaxivity (r_1_) and transverse relaxivity (r_2_) were gained from the slope of the fitting line between the reciprocal of relaxation time and Mn concentration curves (Fig. 4a). We observed that the r_1_ increased while the r_2_ decreased with the decreasing pH value of buffer, which indicates the NCs exhibited a stimuli-response T_1_/T_2_ MRI enhancement. The ratio of r_2_/r_1_ reached as high as 20.6 at pH 7.4, which manifested a more dominant effect of T_2_-enhancement than T_1_ in this condition. The Fe_3_O_4_ in core would generate inhomogeneous magnetic field to influence the T_2_ effect of the water molecules around [7, 37]. The strong local magnetic field generated from the Fe_3_O_4_ ensured the properties of Mn-IOSP NCs for acting as T_2_ contrast agent. The strong magnetic field induced by Fe_3_O_4_ core would disturb the relaxation process of the Mn-porphyrin when they are close enough to generate magnetic coupling [12, 38], and the changes in distance between Mn-porphyrin and Fe_3_O_4_ cores after release attenuated the coupling effects. These factors contributed to the quenching of the T_1_ signal in high pH environment. We hypothesize two synergetic components that led to the abatement of r_2_ after Mn-release. First, the geometrical confinement effect of channels in silica to Mn-porphyrins vanished after release [39–41]. Water molecules inside mesoporous structure of Mn-IOSP NCs can be slowed down because they bind to the slowly moving complexes in the pore or undergo the geometrical restraints of the proximate pore walls [41]. The restriction of water inside the channels might lead to reduction of mobility for the hydration layer, which was conducive to relaxivity [39]. Further, the high surface area-to-volume ratio and water accessibility through the pores allows for an efficient T_1_/T_2_ dual-mode MR contrast agent due to the incorporated complex between H_2_O and Mn-porphyrin. Second, the concentrated Mn paramagnetic centers disappeared after release. When the Mn-porphyrins were loaded in the channels, the substantial quantity of Mn potentially create concentrated Mn paramagnetic centers, leading to an effective magnetic field gradient that dephases water protons and shortens T_2_ in strong fields [39, 40, 42]. However, when the pH decreased, these effects would fade with the release of Mn-porphyrin into solution, which led to a decline of r_2_. The enhancement of the T_1_ relaxivity and the receding T_2_ relaxivity of Mn-IOSP NCs via the decreasing pH value of buffer may be due to the additional releasing of Mn-porphyrin from the mesoporpous. There was no commercial T_1_/T_2_ dual mode contrast agent and commercial Mn-based T_1_ contrast agent yet. Mn_x_O_y_ structures have often been reported as Mn-based CAs [43–46]. The *r*_*1*_ (4.25/mM/s, pH 5.0) and *r*_*2*_ (42.1/mM/s, pH 7.4) of Mn-IOSP was higher than that of the MnO (0.3/mM/s, 3.0 T) and HMnO (1/mM/s, 11.7 T) [43, 44], and was comparable to the T_1_/T_2_ dual mode contrast agent of Fe_3_O_4_@SiO_2_(Gd-DTPA)-RGD NPs (4.2/mM/s for *r*_*1*_ and 17.4/mM/s for *r*_2_, 3.0 T) and manganese oxide/MSNs (3.1 mM/s for r_1_ and 46.1/mM/s for *r*_2_, 3.0 T) which previously reported [18, 47]. Although the r_2_ was not as high as that of the commercial Feridex (108/mM/s, 3.0 T), the Mn-IOSP NCs offer unique features to act as T_1_-positive/T_2_-negative bimodal contrast agents and to some extent could break the restrictions of ordinary one-mode contrast agents. So far as we know, few studies have focused on exploring responsive T_1_/T_2_ dual-mode CAs, much less combination with pH-responsive imaging diagnosis and tumor targeting drug controlled release into one composition. The releasing of Mn-porphyrin actived the contrast ability of Mn-IOSP NCs from single-mode to dual-mode, and guaranteed the capacity for accurate imaging in tumor sites. When the r_2_/r_1_ ratio reduced from 20.6 (pH 7.4) to 7.7 (pH 5.0), the increasing T_1_-contrast enhancement via the releasing of the Mn-porphyrin suggested the Mn-IOSP NCs could act as an ideal T_1_/T_2_ dual-mode contrast agent at acidic environments of tumor areas [14]. These results demonstrated that Mn-IOSP NCs can act as T_1_-positive/T_2_-negative bimodal contrast agents.

For further demonstration of the T_1_ and T_2_-effect and cellular uptake of the Mn-IOSP NCs, in vitro T_1_-weighted and T_2_-weighted MR images of A549 cells were observed after incubated with NCs for 1 h. Quantitative analysis of Fe and Mn in the incubated cells has also been carried out by ICP-MS to help to quantify the cell uptake. The contents of metal in cells after incubation with NCs modified with PAA-cRGD or PAA were analyzed by ICP-MS. The concentrations of both manganese and iron were higher in the cells incubated with the NCs modified with c(RGDyK) than the NCs without c(RGDyK) (Fig. 4b). In addition, the higher of the NCs concentration incubated with cells, the higher the signal intensities of manganese and iron were tested by ICP-MS (Fig. 4c). The results were consilient with the experiments of in vitro cell MRI. As shown in Fig. 4d, the T_1_ and T_2_-weighted MRI contrast effect correlated with the concentration of the Mn-IOSP NCs. The Mn-IOSP NCs displayed an enhancement in the T_1_-weighted MR signal, and a reduction in T_2_-weighted MR signal with the increasing Mn concentration. This confirmed that the Mn-IOSP NCs could be utilized as efficient T_1_ and T_2_ dual-mode contrast agents for A549 tumor cells.

To further validate the ability of Mn-IOSP NCs as T_1_/T_2_ dual-mode MRI agents, we conducted the in vivo MRI of mice which transplanted subcutaneous A549 tumor before and after injecting the Mn-IOSP NCs (200 μL, [Mn] = 2 mM) via the tail vein. As shown in Fig. 4e, the T_1_ and T_2_ relaxation time in mice-transplanted tumor areas was obviously fasted after injection of Mn-IOSP NCs, and T_1_-weighted signal was enhanced whereas T_2_-weighted signal was reduced. Compared to the images without injection of NCs, the most powerful acceleration in tumor signal for T_1_ was 55%, and for T_2_ was 37%, both after 3 h injection of the NCs. T_1_-weighted images took on brighter effects in the region of A549 tumor after injection, whereas darker effects were exhibited in T_2_-weighted images. In addition, after 12 h injection, the signal intensity recovered to some extent. The biodistributions of NPs were evaluated in A549 tumor-bearing mice after 3 h intravenous injection of Mn-IOSP NCs (details in Additional file 1: S7). Both manganese and iron showed significant tumor accumulation (Additional file 1: Fig. S17), which were consistent with the MR images. The Mn-IOSP NCs with appropriate residence time and controlled release performance show the remarkable potential for *in* *vivo* MRI for tumor. The accumulation of Mn-IOSP NCs in tumor might realize through the EPR effect and the targeting effect of c(RGDyK).

These evidences showed that the synthesized Mn-IOSP NCs exhibited highly sensitive MRI contrast function no matter in solution, cells or in vivo, and comprehensive analysis from the information of both T_1_/T_2_ imaging could be conducted, which to some extent breaks the restrictions of ordinary one-mode contrast agents.

### Toxicity

**Fig. 5:**
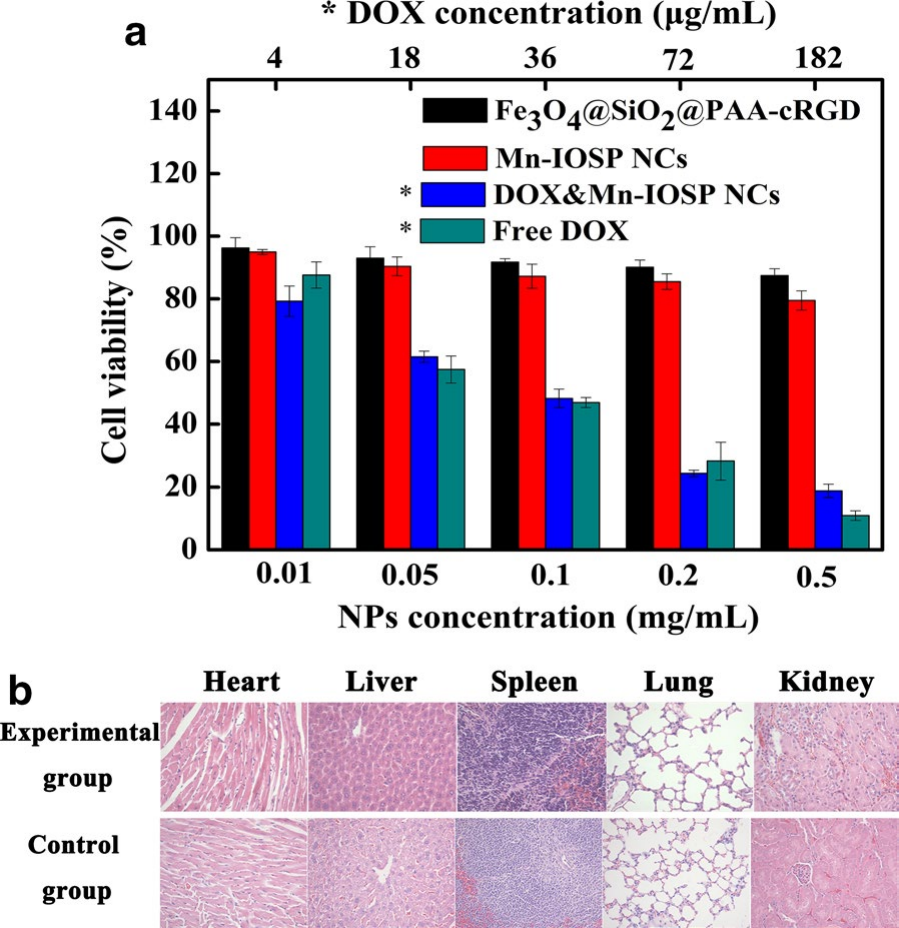
Biotoxicity of nanoparticles: **a** cell viability assays of A549 cells treated with different samples, *for samples calculated by DOX content with the upper abscissa, **b** H&E staining images of histological sections with major organs after 48 h injection of 200 μL Mn-IOSP NCs (experimental group) or 200 μL PBS (control group)

Cytotoxicity of Mn-IOSP NCs in vitro was judged through standard tetrazolium dye (MTT) based colorimetric assay for viability of A549 cell. In the tested range of concentration, the cytoactive of A549 cells had not declined significantly after the incubation with Fe_3_O_4_@SiO_2_@PAA-cRGD NPs and Mn-IOSP NCs (Fig. 5a). The cell viability still remained about 80% after incubated with Mn-IOSP NCs at a very high dose of 500 μg/mL NCs, indicating that the NCs are biocompatible and minimally cytotoxic in the given concentration range. After incubation with DOX-loaded NCs, the cell viabilities significantly decreased compared to the Mn-IOSP NCs and Fe_3_O_4_@SiO_2_@PAA-cRGD NCs of same concentrations. These results demonstrate little toxicity of our NPs and NCs as dual contrast agents. In addition, the effect of DOX-loaded NCs was close to the free DOX compared with the cytotoxicity assay of an equal concentration of DOX alone, for which confirms the feasibility of the NCs as drug carriers systems.

Hematoxylin and Eosin (H&E) staining method was used to assess the toxicity of the developed Mn-IOSP NCs in vivo. Mice treated with Mn-IOSP NCs or PBS (as control) were sacrificed post 48 h i.v. injection. Apparently, we can see from Fig. 5b, no obvious damage or abnormalities can be seen in the major tissue sections such as heart, liver, spleen, lung, and kidney after 48 h i.v. injection of the NCs, which suggests that the Mn-IOSP NCs have good organ compatibility.

## Conclusions

In conclusion, we have developed a multifunctional nanocomposite of Mn-porphyrin&Fe_3_O_4_@SiO_2_@PAA-cRGD for dual-modal bioimaging and drug-loading capacity as a result of our studies. Compared to previous multifunctional contrast agents, the proposed nanocomposite integrates the advantages of synergistically enhancing positive and negative magnetic resonance imaging signals, no matter in solution, cells or in vivo. The r_2_/r_1_ ratio was 20.6 in neutral pH 7.4, which decreased to 7.7 in acidic pH 5.0, suggesting the NCs could act as an ideal T_1_/T_2_ dual-mode contrast agent at acidic environments of tumor. Conjugation with RGD enables the functional imaging probe for high A549 cellular uptake and targeted tumor imaging in vivo. The findings of this study illustrate that Mn-porphyrin&Fe_3_O_4_@SiO_2_@PAA-cRGD is a potentially useful tool for multimodal molecular imaging of cancer cells as well as a drug delivery platform for therapeutic agents.

## Additional file

**Additional file 1.** Additional figures, experimental details and parameters. Part S1: Preparation of Fe_3_O_4_@SiO_2_@PAA-cRGD; part S2, including the Fig. S1–S11: ^1^H NMR, ^13^C NMR, MS, UV spectra and structure for compounds; part S3: Cell culture; part S4: Specific parameters of MR contrast properties test; part S5, including Table S1 and Fig. S12–16: Hydrodynamic diameters of the nanoparticles tested by DLS; part S6: Drug loading and in vitro release; part S7, including Fig. S17: Biodistributions tested by ICP-MS.
