# Supplementary material for: pH-responsive theranostic nanocomposites as synergistically enhancing positive and negative magnetic resonance imaging contrast agents
Source: J Nanobiotechnology. 2018 Mar 27;16:30. doi: 10.1186/s12951-018-0350-5 (PMC5870481; doi:10.1186/s12951-018-0350-5)
Supplement: Supplementary file 1 — Additional file 1. Additional figures, experimental details and parameters. Part S1: Preparation of Fe3O4@SiO2@PAA-cRGD; part S2, including the Fig. S1–S11: 1H NMR, 13C NMR, MS, UV spectra and structure for compounds; part S3: Cell culture; part S4: Specific parameters of MR contrast properties test; part S5, including Table S1 and Fig. S12–16: Hydrodynamic diameters of the nanoparticles tested by DLS; part S6: Drug loading and in vitro release; part S7, including Fig. S17: Biodistributions tested by ICP-MS. [file 12951_2018_350_MOESM1_ESM.docx]

Additional file 1

pH-Responsive Theranostic Nanocomposites as Synergistically Enhancing Positive and Negative Magnetic Resonance Imaging Contrast Agents

Xi Huang, Yaping Yuan, Weiwei Ruan, Lianhua Liu, Maili Liu,

Shizhen Chen^*^, and Xin Zhou^*^

*Address: State Key Laboratory of Magnetic Resonance and Atomic and Molecular Physics,* *Wuhan Institute of Physics and Mathematics, Chinese Academy of Sciences; Wuhan National Laboratory for Optoelectronics, Wuhan 430071, China.*

**Table of Contents**

1. Preparation of Fe_3_O_4_@SiO_2_@PAA-cRGD

2. ^1^H NMR, ^13^C NMR, MS, UV spectra and structure for compounds

3. Cell culture

4. Specific parameters of MR contrast properties test

5. Hydrodynamic diameters of the nanoparticles tested by Dynamic Light Scattering (DLS)

6. Drug loading and *in vitro* release

7. Biodistributions tested by ICP-MS

**S1. Preparation of Fe_3_O_4_@SiO_2_@PAA-cRGD**

In a typical synthesis of Fe_3_O_4_@nSiO_2_^1^, 70 μL of TEOS was added into the mixture of Fe_3_O_4_ (1 mL, dispersed in cyclohexane), NH_3_·H_2_O (150 μL, 28%) and Igepal CO-520 (1 mL). The product was precipitated with excess methanol after 6 h magnetic stirring. After repeated washing and reprecipitation, the as-obtained particles were dispersed in 20 mL water through ultrasonic dispersion. For the synthesis of Fe_3_O_4_@nSiO_2_@mSiO_2_^2^, 10 mL solution of Fe_3_O_4_@nSiO_2_ was well mixed with the 20 mL aqueous solution of CTAC (2.0g) and TEA (0.02g) by stirring for 1 h under 80°C, and the reaction was carried out at 80°C for another 1 h with an addition of 120 μL TEOS into the solution. The particles were obtained by repeated washing and reprecipitation, and the structure-template CTAC was removed by washing with solution of sodium chloride solution (1 wt%, in MeOH) for five times.^3^ The particles were obtained by repeated washing and reprecipitation.

For preparation of Fe_3_O_4_@SiO_2_@PAA-cRGD, we first synthesized PAA-cRGD. NHS (12 mg), EDCI (20 mg) and PAA (400 mg) was dissolved in MES buffer (5 mL, pH 5.5) and reacted for 2 h before c(RGDyK) (10 mg) was dissolved into the mixture with vigorous stirring. Then the pH was adjusted to neutral by PBS buffer, and the solution was incubated under room temperature for 12 h. Excess unreacted peptides, EDCI, NHS and impurities in mixture were removed by dialysis (1000 Da). The Fe_3_O_4_@SiO_2_@PAA-cRGD NPs were synthesized from the change of the interfacial energy among cRGD-PAA, Fe_3_O_4_@SiO_2_ and solvent.^4^ PAA-cRGD (2 mg/mL, 10mL in water) and NH_3_·H_2_O (2 mol/L, 150 μL in water) were mixed with the 10mL aqueous solution of Fe_3_O_4_@SiO_2_ (0.01g) and placed in ultrasonic for 60 min. Then isopropanol (180 mL) was added to the mixture dropwise with stirring. After repeated washing and reprecipitation,the Fe_3_O_4_@SiO_2_@PAA-cRGD NPs were obtained by and dried in a vacuum at 45°C for 12 h. For the synthesis of Fe_3_O_4_@SiO_2_ @PAA, all the steps were the same except the substitution of PAA for PAA-cRGD.

S2 ^1^H NMR, ^13^C NMR, MS, UV spectra and structure for compounds

**5, 10, 15, 20-tetrakis 2-[(4-tert-butyl benzamido)] ethyl carbamate] porphyrin.**





**Fig. S1** Structure of porphyrin compound


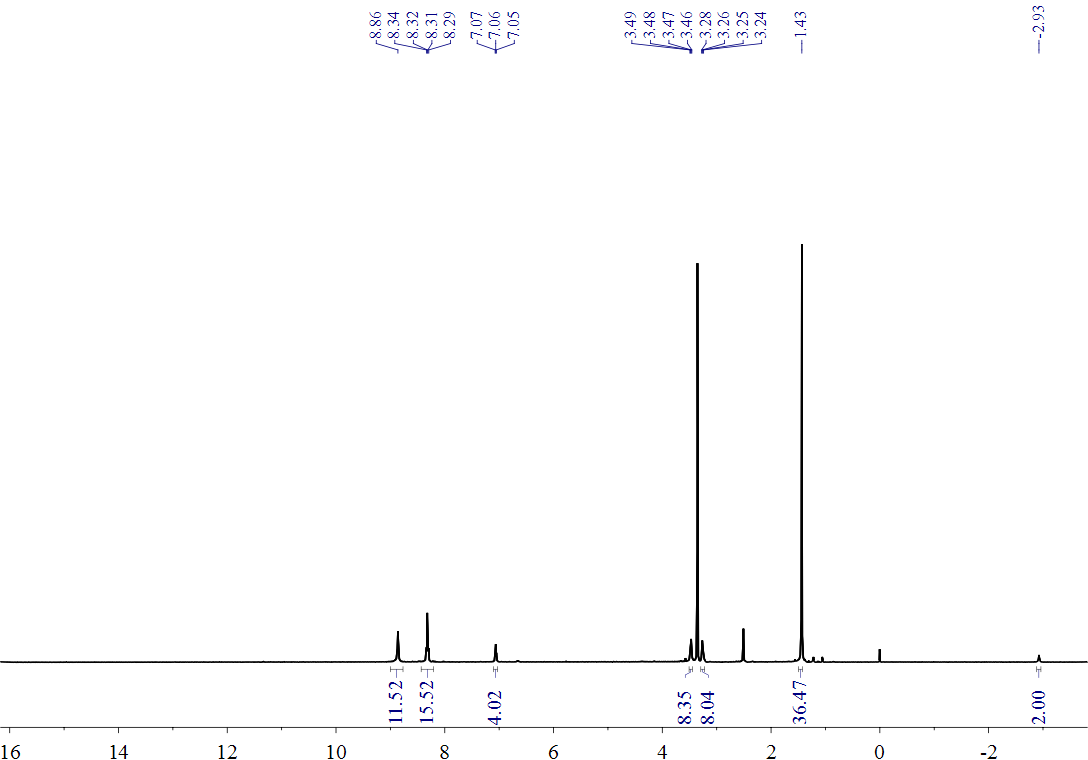


**Fig. S2** ^1^H NMR spectra for porphyrin compound


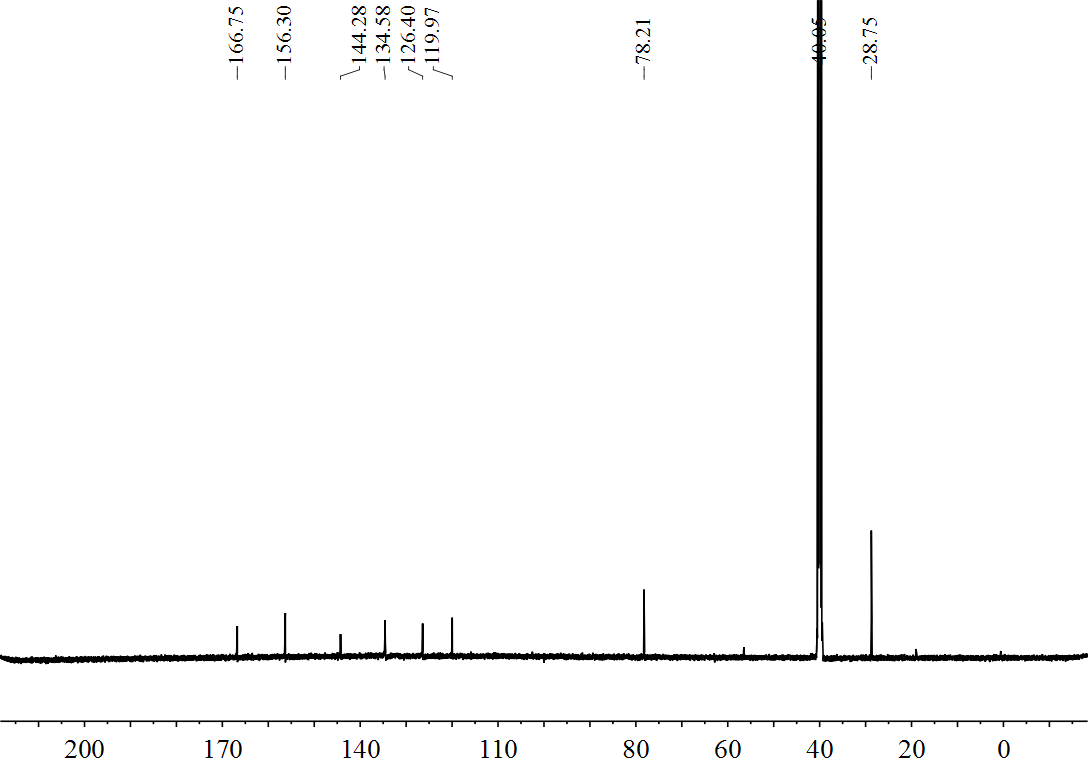


**Fig. S3** ^13^C NMR spectra for porphyrin compound

^1^H NMR: (500MHz) δ (DMSO-*d_6_*) 8.9 (12H, s, β-H+ NH Amide) 8.3 (16H, q, phenyl-H) 7.1 (4H, t, NH-Amide) 3.5 (8H, q, CH2) 3.3 (8H, q, CH2) 1.4 (36H, s, Boc-H) -2.9(2H, s, NH).

^13^C NMR (500 MHz): 167, 156, 144, 135, 126, 120, 78, 40, 28.





**Fig. S4** UV-vis absorption spectra for porphyrin compound

UV (Methanol) Soret-band : 415 nm; Q-bands: 512, 547, 589 and 646 nm.

**Fig. S5** MS spectra for porphyrin compound

Mass Spec: (MALDI MS) Calculated (M) 1358.6488; Observed (M+1) 1359.6594.

**Manganese 5, 10, 15, 20-tetrakis 2-[(4-tert-butyl benzamido) ethyl carbamate] porphyrins (Mn-BOC-porphyrin)**





**Fig. S6** Structure of Mn-BOC-porphyrin compound





**Fig. S7** UV-vis absorption spectra for Mn-BOC-porphyrin compound

UV (Methanol) Soret-band : 467 nm; Q-bands: 564, 598 nm.

**Fig. S8** MS spectra for Mn-BOC-porphyrin compound

Mass Spec: (MALDI MS) Calculated (M) 1411.5712; Observed (M) 1411.5767.

**Manganese porphyrin compounds 1 (Mn-porphyrin)**





**Fig. S9** Structure of Mn-porphyrin compound





**Fig. S10** UV-vis absorption spectra for Mn-porphyrin compound

UV (Methanol) Soret-band : 466 nm; Q-bands: 563, 599 nm.

**Fig. S11** MS spectra for Mn-porphyrin compound

Mass Spec: (MALDI MS) Calculated (M) 1155.2682, (M-4HCl) 1011.3615; Observed (M-4HCl) 1011.3632.

S3 Cell culture

A549 non-small-cell lung cancer cells were cultured in Roswell Park Memorial Institute (RPMI) 1640 medium containing 10% fetal bovine serum and 100 U/mL penicillin and streptomycin. The cells were cultured at 37°C with 5% CO_2。_WI38 human embryonic lung cells were cultured in the same condition, except the substitution of Minimum Essential Medium (MEM) for RPMI-1640.

**S4 Specific parameters of MR contrast properties test**

For *in vivo* MRI, MR scans were performed in axial orientation with the following parameters: 1 mm slice thickness, 3 × 3 cm^2^ field of view (FOV), 256 × 256 matrix. For T_1_-weighted images, TR/TE = 500 ms/11 ms and for T_2_-weighted images, TR/TE = 2500 ms/36 ms. T_1_ relaxation was measured using rapid acquisition with relaxation enhancement (RARE) sequence (TE = 11 ms; TR = 309, 500, 800, 1000, 1500, 2000, 3000 ms); T_2_ relaxation was measured using multi-slice-multi-echo (MSME) sequence (TE = 14, 28, 42, 56, 70, 84, 98, 112, 126, 140 ms; TR = 2500 ms).

For *in vitro* MRI*,* MR scans were performed with following parameters: 4 × 4 cm^2^ FOV, 256 × 256 matrix. For T_2_-weighted images, TR/TE was 8000 ms/75 ms with MSME sequence, and for T_1_-weighted images, TR/TE was 1000 ms/8.5 ms with RARE sequence.

**S5 Hydrodynamic diameters of the nanoparticles tested by DLS**

**Table S1** Hydrodynamic diameters and Zeta potentials of NPs and NCs

| Nanoparticles | Hydrodynamic diameters (nm) | Zeta potentials  (mV) |
| --- | --- | --- |
| Fe_3_O_4_@nSiO_2_ | 113.4±10.4 | -19.8±0.06 |
| Fe_3_O_4_@mSiO_2_ | 140.0±13.9 | -17.0±0.35 |
| Fe_3_O_4_@SiO_2_@PAA-cRGD | 164.2±13.6 | -32.2±0.83 |
| Mn-porphyrin&Fe_3_O_4_@SiO_2_@PAA-cRGD | 166.1±9.17 | -9.63±0.07 |
| DOX&Fe_3_O_4_@SiO_2_@PAA-cRGD | 199.9±10.0 | -19.0±0.30 |

**Fig. S12** Size distribution of Fe_3_O_4_@nSiO_2_ NPs

**Fig. S13** Size distribution of Fe_3_O_4_@mSiO_2_ NPs

**Fig. S14** Size distribution of Fe_3_O_4_@SiO_2_@PAA-cRGD NPs

**Fig. S15** Size distribution of Mn-porphyrin&Fe_3_O_4_@SiO_2_@PAA-cRGD (Mn-IOSP NCs)

**Fig. S16** Size distribution of DOX&Fe_3_O_4_@SiO_2_@PAA-cRGD NCs

**S6 Drug loading and *in vitro* release**

In a typical procedure, 1 mg of Fe_3_O_4_@SiO_2_@PAA NPs (or Mn-IOSP NCs) were dissolved in a mixed solution containing 1 mL water and 9 mL isopropanol, DOX aqueous solution (100 μL, 10 mg/mL) was mixed into the solution before being shaken for 24 h. Finally, the desired NCs were gained through centrifugation separation and the concentrations of DOX in supernatant were determined.

*In vitro* DOX release from Mn-IOSP NCs was tested with dialysis diffusion technique. Equal amount of DOX-loaded Mn-IOSP NCs were dispersed in 1 mL of PBS (pH 7.4, pH 6.5) or acetate buffer (pH 5.0). All the solutions above were transferred into semipermeable dialysis bags (3000 Da) and immersed into 200 mL of corresponding buffer at 37°C with stirring. At predetermined time points, 1 mL of the release medium outside the semipermeable was collected for quantifying the release efficiency of DOX and equal amount of of fresh medium was supplemented.

For investigating the pH-triggered controlled release property of the Mn-porphyrin from the Mn-IOSP NCs, equal amounts of samples were dispersed in different release media to simulate the release behavior in different physiological environments. The supernatant of samples were collected after centrifugation at predetermined time points, and the Mn-porphyrin in the solutions were tested by UV-vis.

S7 Biodistributions tested by ICP-MS

The tissue biodistributions of NPs were evaluated in A549 tumor-bearing mice. The mice were sacrificed after 3 h *i.v.* injection of Mn-IOSP NCs, and the primary organs were hauled out and immersed into normal saline for a moment before blotted with gauze. The tissues were holden in −80 °C overnight and portions of tissues were digested by nitric acid at 100℃ for 1 h. Furthermore, the distributions of Mn and Fe elements in heart, liver, spleen, lung, kidney, and tumor were analyzed by ICP-MS (FLEXAR NEXLON300X) measurement.





**Fig. S17** Biodistributions in primary organs after 3 h *i.v.* injection of Mn-IOSP NCs

**References**

1. Liu ZY, Yi GS, Zhang HT, Ding J, Zhang YW, Xue JM. Monodisperse silica nanoparticles encapsulating upconversion fluorescent and superparamagnetic nanocrystals. Chem Commun. 2008:694-696.

2. Liu JN, Bu JW, Bu WB, Zhang SJ, Pan LM, Fan WP, Chen F, Zhou LP, Peng WJ, Zhao KL, et al. Real-Time In Vivo Quantitative Monitoring of Drug Release by Dual-Mode Magnetic Resonance and Upconverted Luminescence Imaging. Angew Chem Int Edit. 2014;53:4551-4555.

3. Taylor KML, Kim JS, Rieter WJ, An H, Lin WL, Lin WB. Mesoporous silica nanospheres as highly efficient MRI contrast agents. J Am Chem Soc. 2008;130: 2154-2155

4. Li L, Liu C, Zhang LY, Wang TT, Yu H, Wang CG, Su ZM. Multifunctional magnetic-fluorescent eccentric-(concentric-Fe_3_O_4_@SiO_2_)@polyacrylic acid core- shell nanocomposites for cell imaging and pH-responsive drug delivery. Nanoscale. 2013;5:2249-2253.
